# PIK3R1 as a Gastric Cancer Biomarker Linked to CD73^**+**^ Treg-Mediated Immunosuppression

**DOI:** 10.32604/or.2025.069453

**Published:** 2026-01-19

**Authors:** Bu Zou, Yi-En Xu, Hui-Chan He, Zu-Lu Ye, Da-Lei Zhou, Cai-Yun He, Chan Huang

**Affiliations:** 1 Department of Head and Neck, Sun Yat-sen University Cancer Center, State Key Laboratory of Oncology in South China, Collaborative Innovation Center for Cancer Medicine, Guangzhou, 510060, China; 2 Department of Molecular Diagnostics, Sun Yat-sen University Cancer Center, State Key Laboratory of Oncology in South China, Collaborative Innovation Center for Cancer Medicine, Guangzhou, 510060, China; 3 Department of Blood Transfusion, Sun Yat-sen University Cancer Center, State Key Laboratory of Oncology in South China, Collaborative Innovation Center for Cancer Medicine, Guangzhou, 510060, China; 4 Department of Pathology, Sun Yat-sen University Cancer Center, State Key Laboratory of Oncology in South China, Collaborative Innovation Center for Cancer Medicine, Guangzhou, 510060, China

**Keywords:** Cluster of differentiation 73, gastric cancer, immune microenvironment, nomogram, phosphoinositide-3-kinase regulatory subunit 1

## Abstract

**Objectives:**

Gastric cancer (GC) remains a major global health concern, and Phosphoinositide-3-Kinase Regulatory Subunit 1 (PIK3R1), a regulatory subunit of the PI3K signaling pathway, may play a critical yet underexplored role in GC progression. This study aimed to investigate the prognostic significance of PIK3R1 in GC and its association with the tumor immune microenvironment.

**Methods:**

PIK3R1 expression and its clinical relevance were analyzed using datasets from GC patients who underwent gastrectomy, including cohorts from The Cancer Genome Atlas (TCGA) and the Sun Yat-sen University Cancer Center (SYSUCC). Prognostic models integrating PIK3R1 expression with clinical parameters were constructed for both cohorts. The immune microenvironment associated with PIK3R1 expression was assessed through immunohistochemistry and single-cell RNA sequencing. *In vitro* assays were conducted to evaluate the effects of PIK3R1 on GC cell proliferation and migration.

**Results:**

PIK3R1 was significantly overexpressed in GC tissues and was closely associated with aggressive tumor characteristics and poor clinical outcomes. A nomogram combining PIK3R1 expression with clinicopathological features effectively predicted patient prognosis. Knockdown of PIK3R1 in GC cells reduced proliferation and migration *in vitro*. Immunological profiling revealed that high PIK3R1 expression correlated with increased infiltration of forkhead box protein P3 (Foxp3^+^) and cluster of differentiation 73 (CD73^+^) T cells. Patients with low PIK3R1 expression and low CD73^+^ T cell infiltration had significantly better survival.

**Conclusions:**

PIK3R1 overexpression is linked to poor prognosis in GC and influences the extent of immune cell infiltration within the tumor microenvironment. A novel prognostic model integrating PIK3R1 and CD73 expression with clinical parameters was established to stratify GC patients into distinct risk groups, offering potential value for personalized therapeutic strategies.

## 1 Introduction

Gastric cancer (GC) continues to be a common cancer and ranks fifth for incidence and fourth for mortality globally [1]. Despite many therapeutic endeavors, the 5-year survival rate for advanced GC remains relatively low [2]. Furthermore, only a limited number of patients with GC achieve clinical benefits from immunotherapy, especially with immune checkpoint inhibitors (ICIs) against the programmed death-1 (PD-1)/programmed death-ligand 1 (PD-L1) axis [3,4]. To improve the clinical efficacy of immunotherapy, there is a pressing need to identify biomarkers that can predict which GC patients possess intact antitumor immune responses and may benefit from immunotherapeutic strategies. Recent studies, for example, have investigated transcriptional and epigenetic regulators such as metal regulatory transcription factor 1 (MTF1) and m6A-related modulators, which showed strong associations with immune cell infiltration and immune biomarker expression across multiple cancer types, highlighting their potential as an immune-related prognostic biomarker [5,6].

Genetic alterations in key components of the Phosphoinositide-3-Kinase/protein kinase-B (PI3K/AKT) signaling pathway, particularly Phosphatidylinositol 3-kinase catalytic subunit alpha (PIK3CA), phosphatase and tensin homolog (PTEN), and protein kinase-B (AKT), are well known to drive oncogenesis in various tumors [7,8]. Interestingly, emerging evidence has revealed that Phosphoinositide-3-Kinase Regulatory Subunit 1 (PIK3R1), the regulatory subunit of PI3Ks, may also play crucial and context-dependent roles in tumor development [9–11]. For instance, PIK3R1 loss accelerates tumorigenesis in Human epidermal growth factor receptor 2 (HER2)-driven breast cancer models [11]. Conversely, the overexpression of PIK3R1 in hepatocellular cancer promotes tumor progression and metastasis [10].

We previously observed that PIK3R1 is a frequently mutated gene in a subtype of GC that is defined by Epstein-Barr virus (EBV) infection. Similar to EBV-induced nasopharyngeal carcinoma (NPC), EBV-associated gastric cancer also exhibits a highly complex and immunologically active tumor microenvironment, which profoundly influences treatment responses and immune evasion [12–14]. We further observed that the mutation rate of PIK3R1 differed significantly between GC patients with mature and non-mature tertiary lymphoid structures (TLSs) (21% vs. 0%) [15]. Recently, increasing attention has been directed toward components of the tumor microenvironment (TME) such as TLSs and cancer-associated fibroblasts, given their critical roles in modulating antitumor immunity. In particular, TLSs, as ectopic lymphoid aggregates formed in response to immune stimulation, have been linked to favorable immunotherapeutic outcomes [16–20]. These insights raise the important possibility that PIK3R1 may not only contribute to GC progression but also functionally shape the tumor immune microenvironment.

To investigate this, we extended our previous work on pan-cancer PIK3R1 expression patterns, particularly in patients with GC. We combined bulk sequencing data with single-cell (sc) RNA sequencing (RNA-seq) data and focused on the role of PIK3R1 in shaping the tumor microenvironment and its underlying interactions in GC. We also attempted to establish a promising clinicopathological prognostic model consisting of PIK3R1 and other parameters in patients with GC. The findings of this study may facilitate the prediction of immunotherapy outcomes for patients with GC.

## 2 Materials and Methods

### 2.1 Study Samples

We employed a two-stage study design. In the first stage, RNA-seq data for 624 samples, including 414 stomach adenocarcinomas and 210 normal tissue samples, were downloaded from the University of California, Santa Cruz Cancer Genomics Browser (https://xenabrowser.net/datapages/?cohort=TCGA%20TARGET%20GTEx&removeHub=https%3A%2F%2Fxena.treehouse.gi.ucsc.edu%3A443, accessed on 21 November 2025). These data were used to analyze differential gene expression patterns. From these data sets, 375 stomach adenocarcinomas and 32 para-cancer samples were used to analyze the relationship between PIK3R1 expression level and clinicopathological features. We screened all samples with both survival data and PIK3R1 expression data, and finally constructed Kaplan-Meier (KM) survival curves from the TCGA cohort (*n* = 224) and the SYSUCC cohort (*n* = 219) to analyze the overall survival differences between patients with high and low PIK3R1 expression. And DepMap data were used to analyze the PIK3R1 expression in gastric cancer cell lines. In the second stage, the preliminary results were validated by immunohistochemistry (IHC) staining of samples from 230 patients with GC recruited from the Sun Yat-sen University Cancer Center (SYSUCC, Guangzhou, China) from January 2015 to December 2021. The Institutional Review Board of SYSUCC approved this study (SL-B2022-495-01). Informed consent has been obtained from the patients. We used gene ontology (GO) enrichment analysis to functionally annotate differentially expressed genes and Kyoto Encyclopedia of Genes and Genomes (KEGG) pathway enrichment analysis to identify significantly enriched biological pathways. The monocle 2 algorithm was applied to analyze changes in the trajectory of epithelial cells.

### 2.2 The Human Protein Atlas (HPA)

The HPA (www.proteinatlas.org) contains information about the expression profiles of human genes at the protein level in normal and tumor tissues. We employed the HPA to compare PIK3R1 protein expression levels between normal gastric tissue and GC tumor tissue.

### 2.3 Gene Set Enrichment Analysis (GSEA)

The RNA-seq data analysis for GC cell lines was performed by 10K Genomics in Shanghai, China. Differential expression analysis between PIK3R1 high- and low-expression groups was conducted using the ‘DESeq2 (version: 1.40.0)’ package. All genes were subsequently ranked by the absolute value of log fold change (|logFC|) in descending order, and pathway enrichment analysis was carried out using the ‘GSEA (version: 4.10.0)’ package [21].

### 2.4 Single Cell Data Processing

For single-cell sequencing analysis, raw data for GSE150290 (GSM4546300, GSM4546302, GSM4546304, GSM4546306, GSM4546308, GSM4546310, GSM4546312, GSM4546314, GSM4546316, and GSM4546318) were downloaded from the Gene Expression Omnibus portal and the ‘Seurat (version: 4.4.0)’ package was used to process the data in R (version: 4.0.3; R Foundation for Statistical Computing, Vienna, Austria), with R studio (version: 1.3.1903) [22]. The raw GSE150290 data were loaded with Seurat and cells were filtered out if they met one of the following thresholds: (1) lower-than-average variation for all genes; (2) a zero unique molecular identifier (UMI) count for 90% of genes; (3) greater than 10% of the expression level originating from mitochondrial or hemoglobin genes; or (4) a UMI count less than 100 or larger than 20,000. A total of 69,190 cells were included for further analysis, and the variable features of each sample were analyzed after normalization. Data for the total cell population of each sample are summarized in Table S1. We then used the Seurat function “FindIntegrationAnchors” to merge sample files with common anchors among variables (dims = 1:10). Merged data were clustered into 15 and 22 cell populations for T cell and epithelial cell clusters, respectively, using the function “FindClusters” (resolution = 0.5). A reduction of cell clustering was performed using t-distributed stochastic neighbor embedding and principal component analysis. For cell population annotation, we used signatures from the original publication [23]. The signatures of every cell cluster are shown in Table S2.

### 2.5 Immunohistochemistry (IHC) Staining and Assessment

The histopathological diagnosis for each GC case was confirmed by two pathologists. Anti-PIK3R1 (Huabio, Woburn, MA, USA; M1510-2, diluted 1:100), anti-CD8 (ZSGB-BIO, Beijing, China; ZA-0508, diluted 1:200), anti-CD73 (ZSGB-BIO, TA809085S, diluted 1:100), and anti-FOXP3 (Abcam, Cambridge, UK; ab20034, diluted 1:100) were used for IHC staining of consecutive slides. IHC-stained specimens were observed under a 10 × 10 low-power microscope (NIKON Eclipse Ti2-U, Tokyo, Japan). Five high-power fields (10 × 40) were randomly selected to judge the color intensity and count the positive cells. The amount of PIK3R1, CD73, CD8, and FOXP3 was determined by multiplying the positive cell number scores by the staining intensity scores (semi-quantitative integral method). The staining intensity was scored as follows: nut-brown (3 points), light brown-yellow (2 points), light yellow (1 point), and no staining (0 points). The number of positive cells was scored as follows: <5% (0 points), 5%–25% (1 point), 26%–50% (2 points), 51%–75% (3 points), and ≥75% (4 points). The total percentage of positive cells is reported as the average of the five random fields. CD8 and FOXP3 were only expressed in immune cells, while PIK3R1 and CD73 were expressed in both gastric tumor cells and immune cells. Therefore, the expression of PIK3R1 and CD73 was scored by IHC staining in three regions: tumor cells combined with immune cells, only tumor cells, and only immune cells (Table S3). The patients were divided into high and low expression groups for association analysis (Table S3).

### 2.6 Datasets and Model Construction

Based on the RECIST 1.1 criteria, target lesions were evaluated to assess the response and the following definitions were used: complete response (CR), the disappearance of all lesions; partial response (PR), ≥30% decrease in the sum of the longest diameters of target lesions compared with baseline; progressive disease (PD), at least a 20% increase in the sum of the longest diameters of the target lesions with an absolute increase of ≥5 mm; and stable disease (SD), neither PR nor PD [24]. Patients with CR, PR, and SD were classified as “non-progressors” and those with PD were classified as “progressors”.

Within the clinicopathological prognostic model, the expression levels of PIK3R1 and CD73 were converted into categorical variables (high- and low-expression subgroups), as determined by optimal cutoff values. We then derived a nomogram for the constructed PIK3R1-based classifier for GC prognosis. The predictive accuracy and discrimination ability of the nomogram risk scores were determined using a calibration plot and the area under the curve (AUC), respectively. Furthermore, independent validation was conducted using the SYSUCC cohort.

### 2.7 Cell Culture

MKN45 (RRID: CVCL_0434) and HGC27 (RRID: CVCL_1279) cell lines, which have relatively high and low PIK3R1 expression levels, respectively, were purchased from Umine Biotechnology Co., Ltd. (Guangzhou, China). All cell lines were authenticated by STR profiling and flow cytometry and tested negative for mycoplasma contamination. MKN45 and HGC27 were maintained in Roswell Park Memorial Institute (RPMI) 1640 medium (GIBCO, Thermo Fisher Scientific, Waltham, MA, USA, #C11875500BT) with 10% fetal bovine serum (FBS) (GIBCO, Thermo Fisher Scientific, Waltham, MA, USA, #10099141). Knock-down (shRNA-) and overexpressing vectors were constructed and validated (Fig. S1). The sequences for shRNA targeting PIK3R1 and PIK3R1 overexpression are provided in Supplemental File S1. Major reagents, along with catalog IDs and suppliers, are listed in Supplemental File S2. The sequence with the highest efficiency for gene silencing was selected as the short hairpin (sh) RNA and was ligated with the PiggyBac vector backbone. A negative control sequence was used as the scrambled sequence (shNC). ShRNA- and PIK3R1 overexpressing vectors were used for transfecting MKN45 and HGC27 cells, respectively, using the PiggyBac transposase vector. Stable clones of shRNA-PIK3R1-expressing cells and PIK3R1 overexpressing cells were generated by selection using puromycin. Primer sequences for detecting the transcript of PIK3R1 were 5^′^-ACTACTGTAGCCAACAACGGT-3^′^ (forward) and 5^′^-GGTTAATGGGTCAGAGAAGCCA-3^′^ (reverse). The results for shRNA against PIK3R1 and overexpression of PIK3R1 (using quantitative reverse transcription polymerase chain reaction [qRT-PCR]) are shown in Fig. S2A,B.

### 2.8 Quantitative Reverse Transcription Polymerase Chain Reaction (qRT-PCR)

TRIZOL (Thermo Fisher Scientific, Waltham, MA, USA, #15596026CN) was used to extract RNA from HGC27, MKN45 and their stable strains. The concentration and purity of RNA were detected using the one Microvolume UV-Vis Spectrophotometer (NanoDrop, Thermo Fisher, #13400518). Use the reverse transcription kit HiScript IV All-in-One Ultra RT SuperMix for qPCR (Vazyme, Nanjing, China, #R433) for reverse transcription of RNA into cDNA.ChamQ Universal SYBR qPCR Master Mix (Vazyme, Nanjing, China, #Q711) was used for qRT-PCR. The 2^−ΔΔCt^ method was used to analyze the data.

### 2.9 Cell Counting Kit 8 (CCK-8) Assay

MKN45 and HGC27 cells in each group were seeded evenly into 96-well (Corning Incorporated, Corning, NY, USA, #3599) plates at a density of 4 × 10^3^ cells/well. Cells in each well were treated with CCK-8 reagent (Dojindo, Kumamoto, Japan, #CK04-100T) after 0, 24, 48, and 72 h of culture. A microplate spectrophotometer (BioTek, Winooski, VT, USA, Epoch) was used to measure the absorbance at 450 nm.

### 2.10 Clony Formation Assay

Transfected HGC27 cells were counted and plated at a density of 2000 cells per well in the six-well plates (Corning Incorporated, Corning, NY, USA, #3736). And then, the cells were incubated at 37°C with 5% CO_2_ for 7 days. After discarding the medium, it was washed with PBS (ZSGB-Bio, pH: 7.3, Beijing, China, # ZLI-9061) and then fixed with 4% paraformaldehyde and stained with 0.1% crystal violet. Images were captured using an inverted light microscope (NIKON Eclipse Ti2-U).

### 2.11 EdU Staining

Transfected HGC27 cells (4 × 10^4^ cells/well) in 6-well plates (Corning Incorporated, Corning, NY, USA, #3736) were treated with EdU reagent (RIBOBIO, Guangzhou, China, #C10310-1) for 2 h at 37°C. After fixing with 4% formaldehyde, EdU+ cells (labeled red) were monitored and photographed using a fluorescence microscope (Eclipse Ti2-U; NIKON, Tokyo, Japan).

### 2.12 Wound Healing Assay

Transfected HGC27 cells (5 × 10^5^ cells/well) were incubated for 24 h at 37°C in 6-well plates (Corning Incorporated, Corning, NY, USA, #3736). A 100-mL pipette tip was used to make scratches. After washing with PBS (ZSGB-Bio, pH: 7.3, Beijing, China, # ZLI-9061), the cells were cultured for 24 h in a medium supplemented with fetal bovine serum (FBS) (GIBCO, #10099141), and images were captured using an inverted light microscope (NIKON Eclipse Ti2-U) and a real-time dynamic cell imaging analysis system (IncuCyte S3; Sartorius, Göttingen, Germany).

### 2.13 Transwell Assay

Transfected HGC27 cells (7.5 × 10^4^ cells/well) were seeded evenly into the upper chamber (Corning, Inc., Corning, NY, USA, #353097) and the corresponding medium containing 20% FBS (GIBCO, #10099141) was added to the lower chamber. After incubation for 24 h, invading cells were fixed with 4% paraformaldehyde for 30 min at 4°C and stained with 0.1% crystal violet for 10 min at room temperature.

The results were then observed under a microscope (NIKON Eclipse Ti2-U).

### 2.14 Western Blot

HGC27, MKN45, and their stable transgenic cells were lysed on ice for 30 min using RIPA lysate buffer (Beyotime, Shanghai, China, #P0013B), added with protease inhibitors (Beyotime, Shanghai, China, #P1006) and phosphatase inhibitors (Beyotime, Shanghai, China, #P1082). The concentration of the lysates was determined using BCA kits (Beyotime, Shanghai, China, #P0012). The protein was separated by SDS-PAGE, and the protein loading volume of each well was 20 μg. 5% skim milk (Biosharp, Hefei, China, #BS102-500g) was used for 1 h at room temperature to block. The primary antibody was incubated overnight at 4°C. 0.1% PBST (PBS: ZSGB-Bio, pH: 7.3, Beijing, China, #ZLI-9061; tween-20: Hangzhou Fude Biotechnology Co., Ltd., Hangzhou, China, #FD0020) was used to wash for 3 times. The secondary antibody was incubated at room temperature for 40 min. We performed 3 replicates of independent biological samples. Chemiluminescence imaging was performed using a chemiluminescence gel imaging system (Bio-Rad, ChemiDoc MP, Hercules, CA, USA). Analyze the image using ImageJ. The primary antibody is used as follows: anti-AKT (Cell Signaling Technology [CST], Danvers, MA, USA; #4691T, diluted 1:5000); anti-p-AKT (CST, #4060T, diluted 1:5000); anti-PIK3R1 (Proteintech, Rosemont, IL, USA, #60225-1, diluted 1:20,000); anti-CD73 (Proteintech, #15449-1-AP, diluted 1:2000); anti-GAPDH (Proteintech, #60004-1-Ig, diluted 1:25,000).

### 2.15 Statistical Analyses

All statistical tests were conducted using R version 4.0.3. An independent Student’s *t*-test was performed to compare continuous variables between two groups. Categorical data were analyzed using the chi-square test. Multivariate Cox regression was used to screen for significant risk factors and construct a PIK3R1-based classifier for GC prognosis. Correlation coefficients were computed by Spearman’s correlation analysis. Differential expression visualization was implemented using the ‘DESeq2’ package. Receiver operating characteristic curves were plotted using the ‘survival ROC version: 1.0-11’ package. The nomogram and calibration plots were generated using the ‘rms version: 6.7-0’ package. Statistical significance was set at a *p*-value < 0.05.

## 3 Results

### 3.1 Bioinformatic Analysis and Experimental Validation Revealed High Expression Levels of PIK3R1 in GC

**Figure 1:**
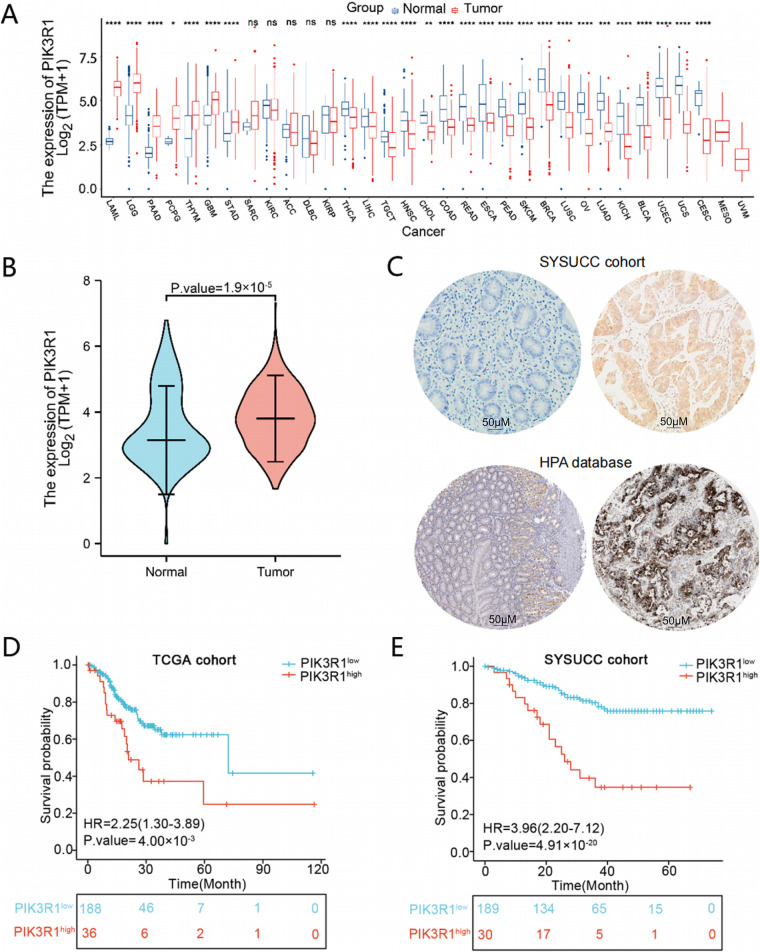
The expression of PIK3R1 in different tumors and normal tissues, and the correlation between PIK3R1 expression and patient prognosis. An-cancer expression pattern of PIK3R1. ****p < 0.0001, ***p < 0.001, **p < 0.01, *p < 0.05, ns: not significant (**A**). Abbreviations: ACC, adrenocortical cancer; BLCA, bladder urothelial carcinoma; BRCA, breast invasive carcinoma; CESC, cervical cancer; CHOL, cholangiocarcinoma; COAD, colon adenocarcinoma; DLBC, large B-cell lymphoma; ESCA, esophageal carcinoma; GBM, glioblastoma mutiforme; HNSC, head and neck squamous cell carcinoma; KICH, kidney chromophobe; KIRC, kidney renal clear cell carcinoma; KIRP, kidney renal papillary cell carcinoma; LAML, acute myeloid leukemia; LGG, lower grade glioma; LIHC, liver hepatocellular carcinoma; LUAD, lung adenocarcinoma; LUSC, lung squamous cell carcinoma; MESO, mesothelioma; OV, ovarian cancer; PAAD, pancreatic cancer; PCPG, pheochromocytoma and paraganglioma; PRAD, prostate adenocarcinoma; READ, rectum adenocarcinoma; SARC, sarcoma; SKCM, melanoma; STAD, stomach adenocarcinoma; TGCT, testicular cancer; THCA, thyroid carcinoma; THYM, thymoma; UCEC, uterine corpus endometrial carcinoma; UCS, uterine adenocarcinoma; UVM, ocular melanoma. mRNA expression levels in GC samples from The Cancer Genome Atlas (TCGA) and GTEx data (*n* (Tumor) = 414; *n* (Normal) = 210) (**B**). Protein expression levels in normal gastric and tumor tissues in samples from the Sun Yat-sen University Cancer Center (SYSUCC) and the Human Protein Atlas databases (**C**). HPA: Normal tissue, https://www.proteinatlas.org/ENSG00000145675PIK3R1/tissue/stomach#img Tumor tissue, https://www.proteinatlas.org/ENSG00000145675-PIK3R1/pathology/stomach+cancer#img. Kaplan-Meier survival curves for PIK3R1 mRNA expression and overall survival (OS) in TCGA (*n* = 224) and SYSUCC cohorts (*n* = 219), respectively (**D**,**E**)

An overview of PIK3R1 transcriptional expression levels was performed in 33 types of cancer. PIK3R1 mRNA was frequently downregulated in most human cancers, while it was significantly upregulated in seven types of cancer (Fig. 1A), indicating a heterogeneous expression pattern and biological role of PIK3R1 across different cancer types. Specifically, PIK3R1 mRNA was significantly upregulated in GC. This finding prompted further investigation into the phenotypic consequences of high PIK3R1 expression levels in GC. An analysis of normalized PIK3R1 mRNA expression data in the TCGA and GTEx databases indicated a significantly higher expression level of PIK3R1 in GC tissues compared to that in normal gastric tissues (number of GC samples/adjacent normal tissues: 414/210, *p* = 1.9 × 10^−5^, Fig. 1B). At the protein level, IHC staining data for GC specimens from the HPA database and SYSUCC cohort confirmed that PIK3R1 was expressed at high levels in tumor cells; however, it was rarely expressed in adjacent normal tissue (Fig. 1C).

### 3.2 Upregulated PIK3R1 mRNA Levels Were Associated with Aggressive Clinicopathological Features and Poor Prognosis in Patients with GC

**Figure 2:**
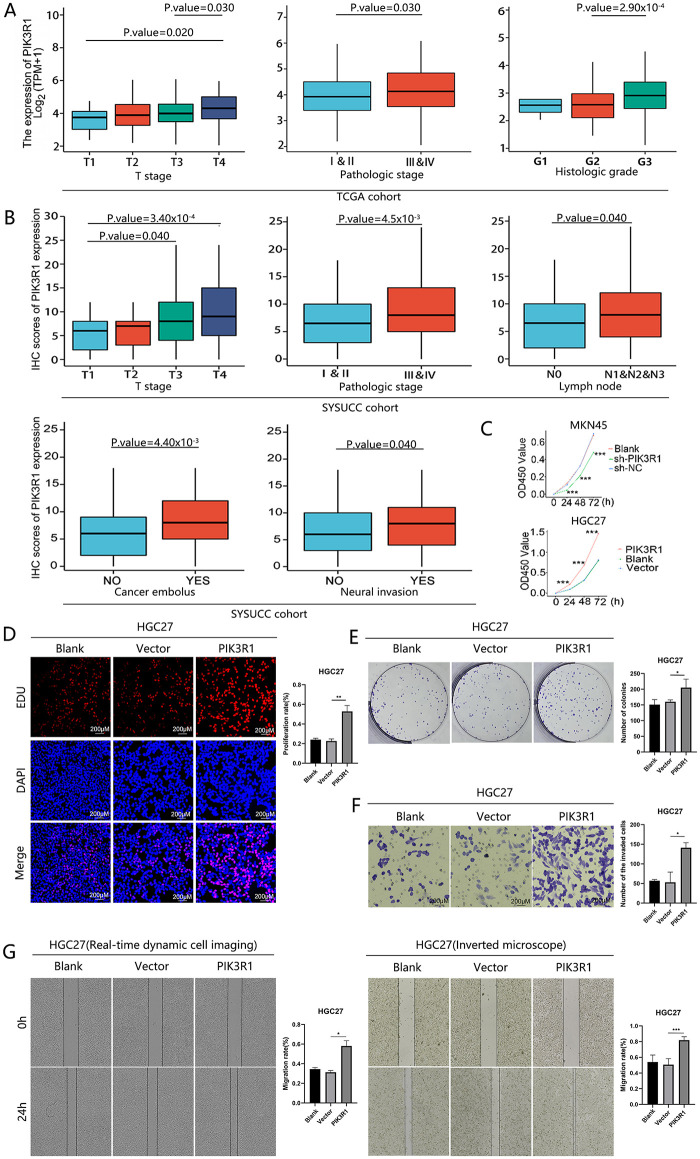
Clinical pathological characteristics and *in vitro* PIK3R1 experiments. PIK3R1 mRNA expression levels were significantly correlated with T stage, histological grade, and pathological stage in the TCGA cohort (*n* =375). PIK3R1 protein expression levels were also strongly correlated with T stage, pathological stage, lymph node metastasis, cancer embolus, and neural invasion in the SYSUCC cohort (*n* = 230) (**A**,**B**). PIK3R1 clearly enhanced gastric cancer (GC) cell proliferation, migration, and invasion. The Cell Counting Kit-8 (CCK8) assay was used to analyze the effects of PIK3R1 overexpression or knockdown on the proliferation of MKN45 or HGC27 cells at the indicated time (**C**). After inducing PIK3R1 overexpression, cell proliferation was tested using 5-Ethynyl-2^′^-deoxyuridine (EdU) staining in HGC27 cells (**D**). Colony-formation assays were used to evaluate the colony-forming capacity of PIK3R1-overexpressing HGC27 cells (**E**). The invasion of HGC27 cells transfected with the PIK3R1-overexpression plasmid was evaluated using transwell assays (**F**). Wound-healing assays showed a change in cell migration capability in PIK3R1-upregulated HGC27 cells (**G**). *p < 0.05, **p < 0.01, ***p < 0.001

The clinical implication of PIK3R1 overexpression in patients with GC was then explored. Preliminary analyses using the TCGA cohort showed that high expression levels of PIK3R1 at the transcript level were positively correlated with advanced histological grade, T, and TNM stages (Table 1 and Fig. 2A). Subsequent validation conducted within the SYSUCC cohort demonstrated that, at the protein level, high expression levels of PIK3R1 in GC were significantly associated with advanced T stage, higher TNM stage, and an increased propensity for lymph node metastasis, intravascular cancer emboli, and neural bundle invasion (Table 1 and Fig. 2B). In addition, we observed that patients with GC with high PIK3R1 expression levels had a shorter overall survival (OS) time (*p* = 4 × 10^−3^ in the TCGA cohort and 4.91 × 10^−20^ in the SYSUCC cohort; Fig. 1D,E). These results suggested that a high PIK3R1 expression level may promote tumor progression and serve as a prognostic marker for patients with GC.

**Table 1:** Correlation between PIK3R1 expression and clinicopathological features in gastric cancer

Clinicopathological features	TCGA cohort		SYSUCC cohort	
	PIK3R1^low^ (188)	PIK3R1^high^ (36)	PIK3R1^low^ (199)	PIK3R1^high^ (31)
Age (Mean ± Standard Deviation)	63 ± 10	63 ± 10	57 ± 11	61 ± 11
	*p* = 0.545		*p* = 0.101	
Sex				
Female	75 (39.9%)	11 (30.6%)	36 (18.1%)	7 (22.6%)
Male	113 (60.1%)	25 (69.4%)	163 (81.9%)	24 (77.4%)
	*p* = 0.385		*p* = 0.727	
Pathology Grade				
G1	2 (1.1%)	0 (0%)	3 (1.5%)	0 (0%)
G2	69 (36.7%)	3 (8.3%)	113 (56.8%)	5 (16.1%)
G3	112 (59.6%)	31 (86.1%)	79 (39.7%)	26 (83.9%)
Gx	5 (2.6%)	2 (5.6%)	4 (2.0%)	0 (0%)
	***p* = 0.002**		***p* = 9.60 × 10** ^**−5**^	
TNM stage				
I + II	102 (54.3%)	13 (36.1%)	88 (44.9%)	8 (25.8%)
III + IV	86 (45.7%)	23 (63.9%)	108 (55.1%)	23 (74.2%)
	***p* = 0.046**		***p* = 0.046**	
M stage				
M0	173 (92.0%)	33 (91.6%)	174 (88.3%)	27 (87.1%)
M1	10 (5.3%)	1 (2.8%)	22 (11.2%)	4 (12.9%)
Mx	5 (2.7%)	2 (5.6%)	1 (0.5%)	0 (0%)
	*p* = 0.583		*p* = 0.795	
N stage				
N0	70 (37.2%)	8 (22.2%)	61 (31.0%)	7 (22.6%)
N1 + N2 + N3	118 (62.8%)	28 (77.8%)	136 (69.0%)	24 (77.4%)
	*p* = 0.083		*p* = 0.343	
T stage				
T1	11 (5.9%)	0 (0%)	42 (21.4%)	1 (3.2%)
T2	38 (20.2%)	5 (13.9%)	30 (15.3%)	0 (0%)
T3	96 (51.0%)	12 (33.3%)	72 (36.7%)	14 (45.2%)
T4	43 (22.9%)	19 (52.8%)	52 (26.5%)	16 (51.6%)
	***p* = 0.004**		***p* = 1.40 × 10** ^**−3**^	
Site				
Stomach Body	45 (23.9%)	5 (13.9%)	66 (57.9%)	12 (52.2%)
Cardia	53 (28.2%)	0 (0%)	17 (14.9%)	4 (17.4%)
Stomach Fundus	24 (12.8%)	10 (27.8%)	5 (4.4%)	1 (4.3%)
Stomach antrum	59 (31.4%)	21 (58.3%)	24 (21.1%)	6 (26.1%)
Stomach Lesser Curvature	1 (0.5%)	0 (0%)	1 (0.9%)	0 (0%)
NOS	6 (3.2%)	0 (0%)	1 (0.9%)	0 (0%)
	***p* = 2.70 × 10** ^**−4**^		*p* = 0.976	

Note: Bold values denote statistical significance at the *p* value < 0.05 level; TCGA, The Cancer Genome Atlas; SYSUCC, Sun Yat-sen University Cancer Center; PIK3R1, Phosphatidylinositol 3-Kinase Regulatory Subunit 1; NOS, Not Otherwise Specified.

### 3.3 PIK3R1 Accelerated the Malignant Progression of GC Cells

Several experiments were performed to test the hypothesis that upregulated PIK3R1 may be involved in GC progression. PIK3R1 was silenced in MKN45 and overexpressed in HGC27 cells (Fig. S2). Subsequently, we studied the influence of upregulated and downregulated PIK3R1 expression on the malignant properties of GC cells. CCK-8 data showed that cell viability was significantly enhanced after the upregulation of PIK3R1 expression, and was markedly diminished after the downregulation of PIK3R1 expression, compared to the viability of control cells (*p* < 0.001; Fig. 2C). Meanwhile, colony-formation assay results indicated that upregulated PIK3R1 expression enhanced the colony-forming capacity of HGC27 cells (Fig. 2D). Similarly, EdU staining revealed the induction of HGC27 cell proliferation mediated by the overexpression of PIK3R1 (*p* < 0.05; Fig. 2E). Moreover, transwell assays showed that PIK3R1 overexpression markedly enhanced the invasiveness of HGC27 cells (*p* <0.05; Fig. 2F). Wound-healing assay data indicated a marked increase in the migration ability of HGC27 overexpressing PIK3R1, compared to those transfected with shRNA and wild-type cells, using conventional live-cell imaging at routine time points and an automated real-time live-cell imaging system (*p* < 0.05; Fig. 2G). These *in vitro* results confirmed that PIK3R1 overexpression accelerated the malignant progression of GC cells.

### 3.4 PIK3R1 Participated in the TGF-***β***/SMAD Signaling Pathway in GC

**Figure 3:**
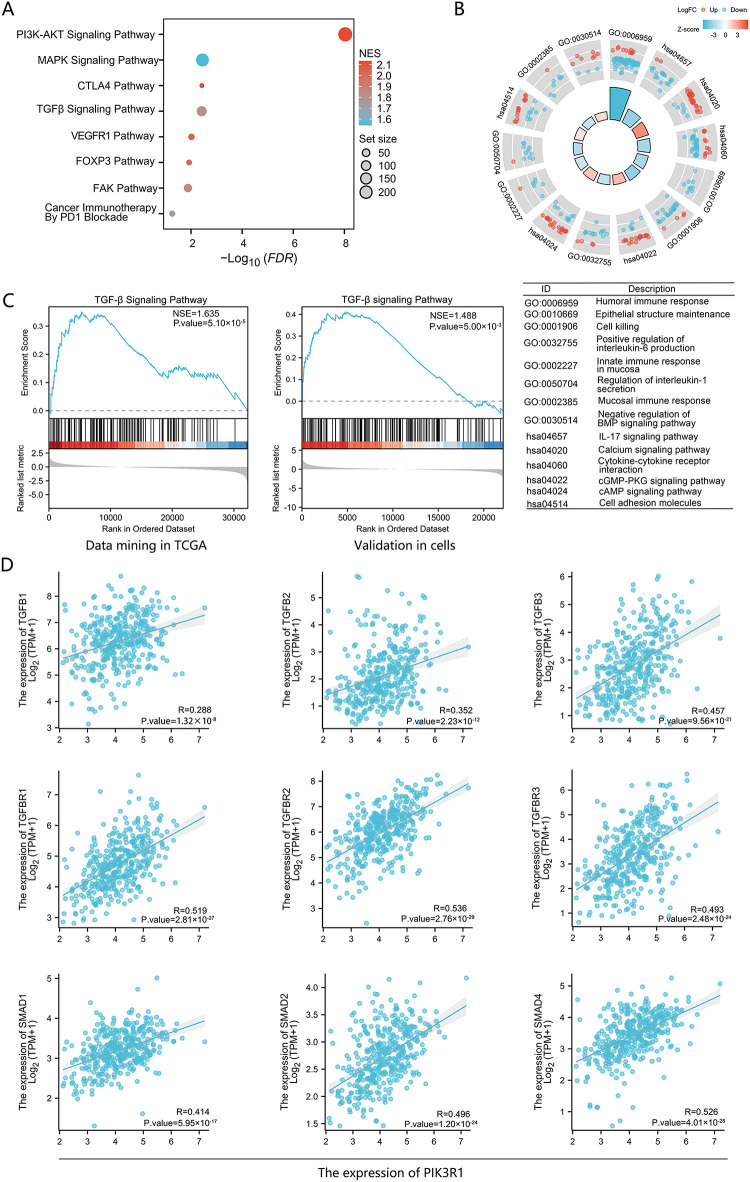
Pathway analysis of the PIK3R1 gene in gastric cancer. Gene set enrichment analysis (GSEA) of differentially expressed genes related to PIK3R1 (**A**). Kyoto Encyclopedia of Genes and Genomes (KEGG) and Gene Ontology (GO) analysis of differentially expressed genes associated with PIK3R1 (**B**). Pathway enrichment analysis of the TCGA cohort and PIK3R1-overexpressing GC cell lines, demonstrating the promotion of the TGF-β signaling pathway (**C**). Positive correlations were observed between the expression levels of PIK3R1 and TGFB1, TGFB2, TGFB3, and their respective receptors, as well as SMAD family genes (SMAD1, SMAD2, and SMAD4; all *p* < 0.05) (**D**)

GSEA analysis of signaling pathway enrichment demonstrated that elevated PIK3R1 expression was significantly enriched in multiple signaling pathways closely associated with tumor development and immune regulation. These pathways were related to PD1, FOXP3, VEGFR1, TGF-β, FAK, and PI3K (Fig. 3A). Gene Ontology (GO) and Kyoto Encyclopedia of Genes and Genomes (KEGG) analyses revealed that the expression level of PIK3R1 was predominantly positively correlated with signaling pathways related to cAMP, cGMP-PKG, cytokine-cytokine interactions, and cell adhesion. Conversely, it was negatively correlated with signaling pathways associated with the humoral immune response involving IL-1, IL-6, and IL-17 (Fig. 3B). Based on the aforementioned enriched pathways, further investigations were performed to determine whether PIK3R1 regulates a crucial cancer-related and immune-regulated pathway, the TGF-β pathway. A positive correlation was observed between PIK3R1 expression levels and the TGF-β signaling pathway when mining TCGA data, and this was validated in GC cell lines (Fig. 3C). Furthermore, PIK3R1 exhibited a positive correlation with the expression levels of TGFB1, TGFB2, and TGFB3 genes and their respective receptors. Additionally, the PIK3R1 expression level was strongly positively correlated with the expression levels of SMAD family gene, including SMAD1, SMAD2, and SMAD4 which are downstream of the TGF-β pathway (Fig. 3D). These results provide evidence suggesting that PIK3R1 participates in tumor immunity and tumor progression signaling networks through enhancement of the TGF-β/SMAD pathway.

### 3.5 PIK3R1 was Associated with the Regulatory T (Treg) Cell-Related Molecules, FOXP3 and CD73 in GC

**Figure 4:**
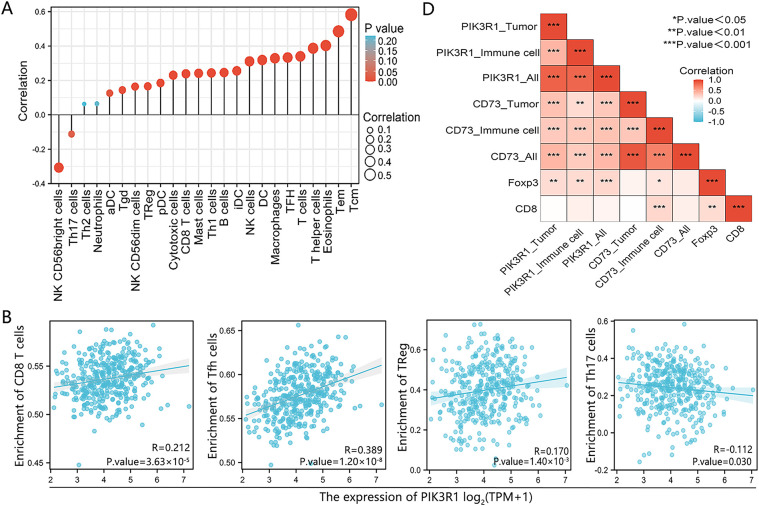

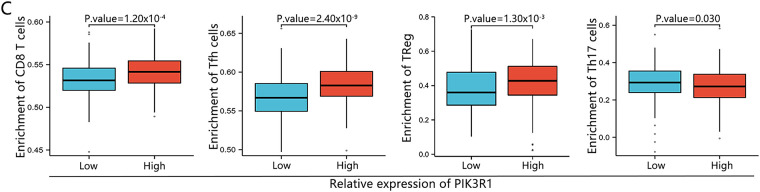
Association between PIK3R1 expression levels and immune cells in GC. The correlation between PIK3R1 expression levels and 24 immune cell types (**A**). Correlation heat map showing the correlation between PIK3R1 expression levels in three different areas and CD73, FOXP3, and CD8 expression levels in GC tissues (**B**). Positive correlations were observed between PIK3R1 expression levels and the number of CD8^+^ T cells, helper T cells, and Treg cells (all *p* < 0.001), while a negative correlation was observed with the number of Th17 cells (*p* = 0.030) (**C**). Correlation heat map showing the correlation between PIK3R1 expression levels in three different areas and CD73, FOXP3, and CD8 expression levels in GC tissues (**D**). *p < 0.05, **p < 0.01, ***p < 0.001

To further analyze the association between PIK3R1 and anticancer immunity, correlations between the expression level of PIK3R1 and the relative abundance of 24 immune cell infiltrates were calculated for GC using TCGA data (Fig. 4A). PIK3R1 levels were positively correlated with the abundance of multiple T cell subsets, including CD8^+^ T cells and Treg cells (Fig. 4B, all *p* < 0.05). An increase in CD8^+^ T cell and Treg cell infiltration was observed in the PIK3R1^high^ group compared to that in the PIK3R1^low^ group (Fig. 4C, both *p* < 0.001). In a survey of other biomarkers of the above-mentioned immune cells, we noticed positive associations between PIK3R1 expression levels and sets of CD8^+^ T cell markers (CD8A and CD8B), immunosuppressive cell markers, Treg cell markers (STAT5B, FOXP3, CD73, and CD4) and exhausted CD8^+^ T cells markers (PD-1, CTLA4, TIM-3, TIGIT, and BTLA) (Table 2). IHC analysis of paraffin-embedded GC tumor sections from the SYSUCC cohort confirmed that the expression of FOXP3 and CD73 were significantly higher in tumor cells from the PIK3R1^high^ group than those from the PIK3R1^low^ group (all *p* < 0.05, Table S3), which also showed positive correlations (Fig. 4D). However, no statistical difference was observed in the expression level of CD8 between PIK3R1^high^ and PIK3R1^low^ groups (Table S3). Knockdown of PIK3R1 in MKN45 cells showed that PIK3R1 silencing reduced CD73 expression. Meanwhile, overexpression of PIK3R1 in HGC27 cells was shown to upregulate CD73 expression (Fig. S2C), and the unedited Western blots are provided in Fig. S4. These results indicated that PIK3R1 may play an important regulatory role in FOXP3- and CD73-related Treg cells.

**Table 2:** Correlation analysis between PIK3R1 and related genes of immune cells

Description	Gene Markers	Cor. (95%CI)		*p* Value
CD8^+^ T cell	CD8A	0.32	0.23–0.41	**2.48 × 10** ^**−10**^
	CD8B	0.17	0.07–0.26	**1.20 × 10** ^**−3**^
Treg cell	FOXP3	0.30	0.21–0.39	**1.89 × 10** ^**−9**^
	CD73(NT5E)	0.43	0.35–0.51	**1.47 × 10** ^**−18**^
	STAT5B	0.68	0.62–0.73	**1.72 × 10** ^**−52**^
	TGFB1	0.29	0.20–0.38	**7.11 × 10** ^**−9**^
	CD25 (IL2RA)	0.38	0.29–0.47	**1.47 × 10** ^**−14**^
	CD4	0.46	0.37–0.53	**7.71 × 10** ^**−21**^
exhausted CD8^+^ T cell	PD-1(PDCD1)	0.23	0.13–0.32	**7.09 × 10** ^**−6**^
	CTLA4	0.28	0.18–0.37	**3.50 × 10** ^**−8**^
	LAG3	0.16	0.06–0.26	**2.00 × 10** ^**−3**^
	TIM-3(HA VCR2)	0.42	0.33–0.50	**1.54 × 10** ^**−17**^
	BTLA	0.35	0.26–0.44	**1.76 × 10** ^**−12**^
	TIGIT	0.35	0.26–0.44	**1.22 × 10** ^**−12**^

Note: Bold values denote statistical significance at the *p* value < 0.05 level, CI, Confidence Interval.

### 3.6 Landscape of PIK3R1 Expression through scRNA-Seq Analysis in GC

**Figure 5:**
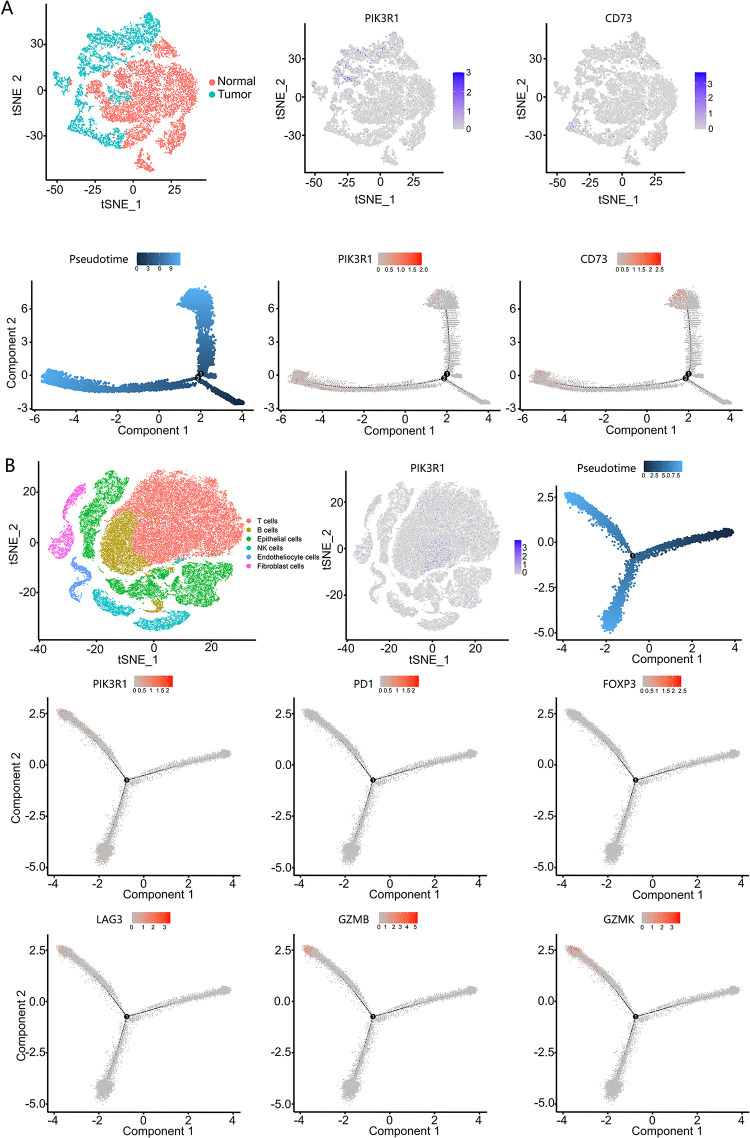
Single-cell RNA sequencing analysis of PIK3R1 and immunosuppressive factor expression levels in gastric epithelial cells, as well as T cells. PIK3R1 and CD73 were mainly expressed in malignant epithelial cells and at the same stage in the dynamic trajectory of epithelial cell development (**A**). PIK3R1 was also expressed in the T cells of GC tissues, and an increased tendency for the expression of PIK3R1 with FOXP3, GZMB, GZMK, PD1, and LAG3 was observed at the same stage of the T cell development trajectory (**B**)

We employed phenotype-specific gene signatures for different cell types from Zhang M et al. [23] to computationally dissect the transcriptional heterogeneity of primary gastric adenocarcinoma based on scRNA-seq profiles (Table S2). Epithelial cells were divided into benign and malignant cells. The monocle 2 algorithm [25] was applied to analyze changes in the trajectory of epithelial cells. In the tree structure diagram, PIK3R1 and CD73 genes were mainly expressed in malignant epithelial cells during the development of malignant cells from benign cells, showing an increasing trend (Fig. 5A). We also analyzed the relationship between PIK3R1 and immunosuppressive molecules secreted by T cells at different stages of GC. Accordingly, molecules reported to be related to immunosuppressive effects, namely, FOXP3, LAG3, PD1, GZMB, and GZMK, were all located at the same stage of the T-cell developmental trajectory with PIK3R1 expression (Fig. 5B). This suggests that PIK3R1 expression occurs predominantly in the immunosuppressive microenvironment.

### 3.7 Nomogram Development for Predicting the Prognosis of Patients with GC

Survival analysis based on a single factor showed that patients with high CD73 and PIK3R1 expression levels and low CD8 expression levels may experience poorer OS (Table S4 and Fig. S3). We next explored their combined effect on patient prognosis. The PIK3R1^low^-CD8^high^ and PIK3R1^low^-CD8^low^ subgroups showed prolonged OS compared to the PIK3R1^high^-CD8^low^ subgroup (TCGA: *p* = 0.004, Fig. S3B; SYSUCC: *p* = 1.86 × 10^−6^, Fig. S3F), while the PIK3R1^high^-CD8^high^ subgroup did not show a favorable prognosis. This finding may indicate that PIK3R1 overexpression inhibits the cytotoxic function of CD8^+^ T cells in patients with GC. Additionally, PIK3R1^high^-CD73^high^ showed a worse OS than the other subgroups (TCGA: *p* = 7.42 × 10^−5^, Fig. S3D; SYSUCC: *p* = 2.66 × 10^−15^, Fig. S3H), which suggested that PIK3R1 may have a synergetic effect with CD73 in patients with GC.

**Figure 6:**
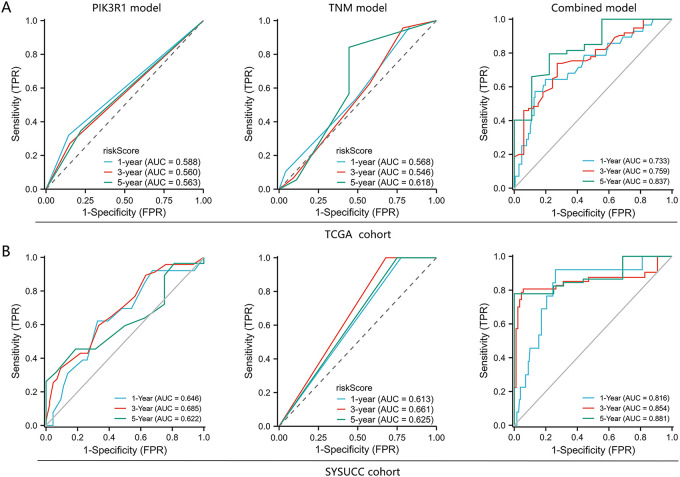

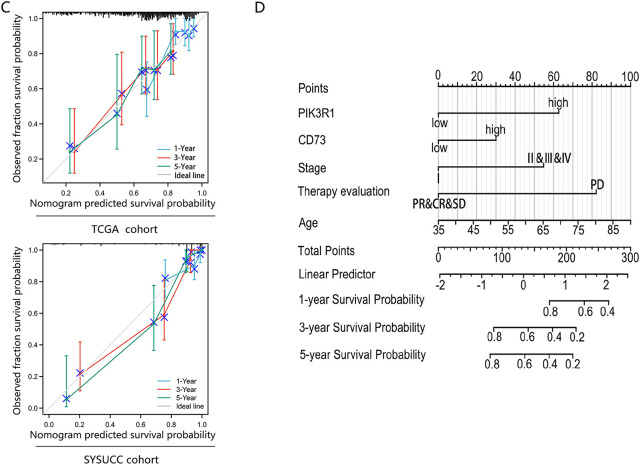
Predictive performance of an integrated model of PIK3R1 for the prognosis of GC compared with TNM staging and PIK3R1 expression levels alone. Receiver operating characteristic curves (ROCs) of three models for predicting OS in patients with GC in the TCGA (*n* = 224) and SYSUCC cohorts (*n* = 217) (**A**,**B**). Calibration curves of the integrated model for predicting the OS of patients with GC in the TCGA and SYSUCC cohorts (**C**). Nomogram for predicting OS (**D**)

We further constructed a model for predicting patient prognosis using multivariate Cox regression analysis, combining PIK3R1 and CD73 with factors including age, TNM stage, and therapy evaluation (Table S4). The AUC value of this combined model was greater than 0.7 in the TCGA cohort and greater than 0.8 in the SYSUCC cohort (Fig. 6A,B), which significantly outperformed the models including PIK3R1 alone or TNM stage (Table 3). The calibration curve revealed excellent agreement between estimates derived from the model and actual probabilities (Fig. 6C). The nomogram-generated scores for each patient are depicted in Fig. 6D, with a high score indicating a high risk of death. To assess the accuracy of the nomogram, patients were classified into low-risk, intermediate-risk, high-risk, or very high-risk groups according to the quartile of the predicted score (Table 4). A greater actual number of deaths was observed for patients with a higher risk.

**Table 3:** Performance of prediction models for GC prognosis

Dataset	AUC (95% CI)	Cutoff	Sen	Spe	PPV	NPV
**TCGA cohort**						
PIK3R1 model						
1 year	0.55 (0.43–0.70)	21.39	0.47	0.69	0.19	0.89
3 years	0.60 (0.48–0.72)	20.07	0.49	0.70	0.51	0.68
5 years	0.66 (0.44–0.88)	7.75	0.94	0.44	0.61	0.89
***p* = 4.90 × 10** ^**−2**^						
TNM model						
1 year	0.57 (0.47–0.67)	21.39	0.47	0.69	0.19	0.89
3 years	0.55 (0.43–0.67)	20.07	0.49	0.70	0.51	0.68
5 years	0.62 (0.38–0.86)	7.75	0.94	0.44	0.61	0.89
***p* = 3.30 × 10** ^**−2**^						
Combined model						
1 year	0.73 (0.63–0.84)	1.52	0.64	0.80	0.34	0.93
3 years	0.76 (0.66–0.86)	0.85	0.74	0.73	0.64	0.81
5 years	0.84 (0.70–0.97)	0.78	0.80	0.78	0.76	0.81
***p* = 1.01 × 10** ^**−6**^						
**SYSUCC cohort**						
PIK3R1 model						
1 year	0.65 (0.50–0.80)	9.00	0.62	0.67	0.12	0.96
3 years	0.69 (0.59–0.78)	5.00	0.89	0.37	0.33	0.91
5 years	0.62 (0.48–0.77)	11.00	0.46	0.81	0.51	0.77
***p* = 6.14 × 10** ^**−7**^						
TNM model						
1 year	0.61 (/)	1.92 × 10^−7^	1.00	0.23	0.08	1.00
3 years	0.66 (/)	1.92 × 10^−7^	1.00	0.32	0.33	1.00
5 years	0.63 (/)	1.92 × 10^−7^	1.00	0.25	0.37	1.00
***p* = 5.00 × 10** ^**−3**^						
Combined model						
1 year	0.83 (0.70–0.93)	1.23	0.92	0.79	0.23	0.99
3 years	0.85 (0.76–0.95)	0.41	0.90	0.91	0.77	0.96
5 years	0.88 (0.80–0.96)	0.41	0.89	1.00	1.00	0.95
***p* = 5.75 × 10** ^**−19**^						

Note: Bold values denote statistical significance at the *p* value < 0.05 level; GC, Gastric cancer; TCGA, The Cancer Genome Atlas; SYSUCC, Sun Yat-sen University Cancer Center; AUC, area under the curve; CI, Confidence Interval; Sen, Sensitivity; Spe, Specificity; PPV, Positive Predictive Value; NPV, Negative Predictive Value; /, data not available or not analyzable.

**Table 4:** Number of events in the nomogram-defined risk groups

Dataset	Risk group 1	Risk group 2	Risk group 3	Risk group 4
TCGA cohort (*n* = 224)	*n* = 56	*n* = 56	*n* = 56	*n* = 56
	9 (16.07%)	8 (14.29%)	16 (28.57%)	31 (55.36%)
Validation cohort (*n* =217)	*n* = 54	*n* = 54	*n* = 54	*n* = 55
	1 (1.85%)	3 (5.56%)	5 (9.26%)	40 (74.07%)

## 4 Discussion

In this study, we elucidated a multifaceted role for PIK3R1 in the progression and immune modulation of gastric cancer (GC), providing new insights into its potential oncogenic function within the unique context of GC. While PIK3R1 has been implicated as a tumor suppressor in cancers like breast and hepatocellular carcinoma, our findings indicate that it may act as an oncogene in GC. This disparity across cancer types suggests that the role of PIK3R1 is heavily context-dependent, shaped by the unique molecular environment of each cancer type.

PI3K signaling plays a role in tumorigenesis, while PIK3R1, a critical regulatory subunit of PI3K, may exert both positive and negative effects on PI3K signaling [10,11,26]. Our results showed that increased expression levels of PIK3R1 correlate with aggressive characteristics, including a poor prognosis in patients with GC. Furthermore, increased expression levels of PIK3R1 in GC at both mRNA and protein levels in two independent cohorts and confirmed *in vitro* experiments are consistent with the notion that it has oncogenic properties. Our previous study showed that loss-of-function mutations in PIK3R1 were enriched in a GC subtype that is typically associated with a better prognosis, indirectly suggesting a possible oncogenic effect of increased PIK3R1 expression levels in GC [12]. PIK3R1 has also been reported to exert tumor suppressor properties in many other cancer types, such as breast cancer and hepatocellular carcinoma [26]. The reason for this discrepancy is unknown, though it likely stems from the discrepant gene expression pattern of PIK3R1 among different types of cancers. However, in the case of GC, data from this study indicate that PIK3R1 may be an oncogene.

We aimed to define the mechanisms of action of PIK3R1 in the pathogenesis of GC. An initial hint of the regulatory role of PIK3R1 in immunosuppressive microenvironments came from findings that the expression level of PIK3R1 was associated with the levels of T cells, especially the FOXP3^+^ Treg cell subpopulation, and with the levels of other immunosuppressive factors. Using scRNA-seq analysis, we observed that PIK3R1 was co-expressed with a series of immunosuppressive molecules, such as CD73, FOXP3, GZMB, and GZMK, during the T cell developmental trajectory. We also noted that PIK3R1 overexpression activated the TGF-β/SMAD signaling pathway in a TCGA cohort and in GC cell lines. Furthermore, our study revealed a close relationship between PIK3R1 and the immunosuppressor, CD73, and their combined effect resulted in worse OS. Based on the tree structure diagram of the scRNA sequencing analysis, PIK3R1 and CD73 were also found to exhibit a synchronous expression pattern during the development of malignant cells from benign cells. CD73 is an ecto-enzyme that can act as a switch for the adenosine-related signaling pathway, which can promote tumor growth and metastasis [27–29]. At the molecular level, ample evidence suggests that TGF-β dysregulation has a causal role in many forms of cancer [30]. Pleiotropic TGF-β can directly regulate the generation of CD73-regulated immune cells, thus contributing to the tumor microenvironment [31]. TGF-β has been reported to markedly suppress the cytotoxic function of CD8^+^ T cells and affect CD4^+^ T cell differentiation and function by inducing Treg cell conversion [32,33]. Among T cells, CD73 is expressed most abundantly by Treg cells and it is responsible for a substantial component of the suppressive capabilities of Treg cells [34,35]. This molecule may inhibit the function of protective immune cells, such as CD8^+^ T cells and natural killer cells, and simultaneously increase the abundance of immunosuppressive cells, such as Treg cells, thus promoting immune escape [36–38]. The defined expression of CD73 by Treg cells in immunosuppressive environments indicates that it is a checkpoint inhibitor [34]. We observed that over-expressing PIK3R1 may have a synergetic effect with CD73^+^ Treg cells to enhance immunosuppression in GC. These findings provide novel insights into the role of increased PIK3R1 expression in the immunosuppressive microenvironment of GC.

Notably, the PI3K signaling pathway, in which PIK3R1 is a key component, may interact with several immune checkpoint inhibitors currently used in clinical practice. The PI3K/AKT/mTOR axis can regulate the expression and stability of PD-L1, and dysregulation of PIK3R1 may enhance immune evasion by upregulating PD-L1 expression [39,40]. PI3K is closely related to the tumor microenvironment [41–43]. In various solid tumor models, PI3K inhibitors have been shown to potentiate the antitumor effects of PD-1 or CTLA-4 inhibitors by reversing the immunosuppressive tumor microenvironment through mechanisms such as downregulating PD-L1 expression, promoting CD8^**+**^ T cell activation, and reducing the activity of regulatory T cells (Tregs) and myeloid-derived suppressor cells (MDSCs) [44–46]. Additionally, alterations in the PI3K pathway, including mutations in PIK3R1 and PIK3CA, have been associated with higher response rates to immunotherapy across multiple cancer types, including gastric cancer [47,48]. The PI3K pathway may also influence T cell function by modulating other immune checkpoints such as CTLA-4 and LAG-3, suggesting that targeting PIK3R1 could offer synergistic therapeutic potential when combined with PD-1/PD-L1 or CTLA-4 inhibitors [44,46,49]. Therefore, PIK3R1 may serve not only as a critical regulator of the immunosuppressive microenvironment but also as a promising therapeutic target in future combination strategies involving immune checkpoint blockade [50].

Additionally, an easy-to-use nomogram model based on PIK3R1 and CD73 expression levels, and several clinicopathological factors, exhibited prognostic significance. In this study, in addition to PIK3R1 and CD73, age, TNM staging, and therapy evaluation were independent prognostic factors for OS. These factors were all included in the nomogram developed to predict OS, which showed better predictive accuracy and prognostic value than the conventional TNM staging system and PIK3R1 alone. These integrated models were developed using the TCGA cohort and were validated in the SYSUCC cohort, and they may be valuable tools for predicting the prognosis of GC. Using the nomogram-defined score, we successfully classified patients into low-, intermediate-, high-, and very-high-risk groups. These proposed risk groups significantly predicted the risk of GC. They may be further optimized to predict immunotherapy efficacy and facilitate population screening in future studies.

It is important to emphasize that our study was not limited to identifying PIK3R1 as a potential prognostic biomarker in the TCGA cohort; we further validated its association with survival outcomes in an independent Chinese patient cohort, thereby enhancing the robustness and clinical relevance of our findings. Nevertheless, this study has certain limitations. First, although we observed a strong correlation between PIK3R1 and CD73 expression, suggesting a possible regulatory relationship, the precise molecular mechanisms through which PIK3R1 may regulate CD73 expression remain to be elucidated. Second, we have not yet conducted a comparative analysis of the prognostic value of CD73 relative to existing immune checkpoints such as PD-L1 and CTLA-4. Future studies should explore their relative and complementary prognostic potential to inform the development of more precise immunotherapeutic strategies.

In summary, we initially provided important clues for the regulatory role of high PIK3R1 expression levels in the progression of GC and the promotion of the immunosuppressive microenvironment, especially increased FOXP3^+^ and CD73^+^ Treg cell infiltration and overactivation of TGF-β/SMAD signaling. We also revealed a synchronous expression pattern of PIK3R1 with a series of immunosuppressive molecules during the development of malignant cells from benign cells. The integrated nomogram, which combined PIK3R1 and CD73 expression levels with other clinicopathological factors, achieved excellent discriminative performance and significantly outperformed TNM staging alone for predicting GC prognosis.

## 5 Conclusions

PIK3R1 not only acts as a prognostic factor to predict the clinical outcomes of patients with GC, but may also be important for the development of novel immunotherapeutic strategies based on data-supported biomarkers to refine patient selection for clinical trials.

## Supplementary Materials



## Data Availability

The data used in the preparation of this manuscript are available from the authors on request.

## Supplementary Materials

The supplementary material is available online at https://www.techscience.com/doi/10.32604/or.2025.069453/s1.

## References

[1] Sung H, Ferlay J, Siegel RL, Laversanne M, Soerjomataram I, Jemal A, et al. Global cancer statistics 2020: GLOBOCAN estimates of incidence and mortality worldwide for 36 cancers in 185 countries. CA Cancer J Clin. 2021;71(3):209–49. doi:10.3322/caac.21660; 33538338

[2] Smyth EC, Gambardella V, Cervantes A, Fleitas T. Checkpoint inhibitors for gastroesophageal cancers: dissecting heterogeneity to better understand their role in first-line and adjuvant therapy. Ann Oncol off J Eur Soc Med Oncol. 2021;32(5):590–9. doi:10.1016/j.annonc.2021.02.004; 33609722

[3] Fuchs CS, Doi T, Jang RW, Muro K, Satoh T, Machado M, et al. Safety and efficacy of pembrolizumab monotherapy in patients with previously treated advanced gastric and gastroesophageal junction cancer: phase 2 clinical KEYNOTE-059 trial. JAMA Oncol. 2018;4:e180013. doi:10.1001/jamaoncol.2018.0013; 29543932 PMC5885175

[4] Shitara K, Van Cutsem E, Bang YJ, Fuchs C, Wyrwicz L, Lee KW, et al. Efficacy and safety of pembrolizumab or pembrolizumab plus chemotherapy vs. chemotherapy alone for patients with first-line, advanced gastric cancer: the KEYNOTE-062 phase 3 randomized clinical trial. JAMA Oncol. 2020;6(10):1571–80. doi:10.1001/jamaoncol.2020.3370; 32880601 PMC7489405

[5] Zhang C, Wang S, Tang H, Lai R, Cai Q, Su Y, et al. Prognostic and immunological role of cuproptosis-related gene MTF1 in pan-cancer. J Cancer. 2024;15(17):5786–809. doi:10.7150/jca.98749; 39308676 PMC11414622

[6] Wang J, Zhao G, Zhao Y, Zhao Z, Yang S, Zhou A, et al. N^6^-methylation in the development, diagnosis, and treatment of gastric cancer. J Transl Int Med. 2024;12:5–21. doi:10.2478/jtim-2023-0103; 38525439 PMC10956730

[7] Fattahi S, Amjadi-Moheb F, Tabaripour R, Ashrafi GH, Akhavan-Niaki H. PI3K/AKT/mTOR signaling in gastric cancer: epigenetics and beyond. Life Sci. 2020;262:118513. doi:10.1016/j.lfs.2020.118513; 33011222

[8] Ke M, Zhu H, Lin Y, Zhang Y, Tang T, Xie Y, et al. Actin-related protein 2/3 complex subunit 1B promotes ovarian cancer progression by regulating the AKT/PI3K/mTOR signaling pathway. J Transl Int Med. 2024;12:406–23. doi:10.2478/jtim-2024-0025; 39360160 PMC11444474

[9] Han MW, Ryu IS, Lee JC, Kim SH, Chang HW, Lee YS, et al. Phosphorylation of PI3K regulatory subunit p85 contributes to resistance against PI3K inhibitors in radioresistant head and neck cancer. Oral Oncol. 2018;78:56–63. doi:10.1016/j.oraloncology.2018.01.014; 29496059

[10] Paskeh MDA, Ghadyani F, Hashemi M, Abbaspour A, Zabolian A, Javanshir S, et al. Biological impact and therapeutic perspective of targeting PI3K/Akt signaling in hepatocellular carcinoma: promises and challenges. Pharmacol Res. 2023;187:106553. doi:10.1016/j.phrs.2022.106553; 36400343

[11] Thorpe LM, Spangle JM, Ohlson CE, Cheng H, Roberts TM, Cantley LC, et al. PI3K-p110α mediates the oncogenic activity induced by loss of the novel tumor suppressor PI3K-p85α. Proc Natl Acad Sci U S A. 2017;114(27):7095–100. doi:10.1073/pnas.1704706114; 28630349 PMC5502636

[12] He CY, Qiu MZ, Yang XH, Zhou DL, Ma JJ, Long YK, et al. Classification of gastric cancer by EBV status combined with molecular profiling predicts patient prognosis. Clin Transl Med. 2020;10(1):353–62. doi:10.1002/ctm2.32; 32508039 PMC7240851

[13] Liu H, Tang L, Li Y, Xie W, Zhang L, Tang H, et al. Nasopharyngeal carcinoma: current views on the tumor microenvironment’s impact on drug resistance and clinical outcomes. Mol Cancer. 2024;23(1):20. doi:10.1186/s12943-023-01928-2; 38254110 PMC10802008

[14] Zhao X, Huang X, Dang C, Wang X, Qi Y, Li H. The Epstein-Barr virus-miRNA-BART6-5p regulates TGF-β/SMAD4 pathway to induce glycolysis and enhance proliferation and metastasis of gastric cancer cells. Oncol Res. 2024;32(5):999–1009. doi:10.32604/or.2024.046679; 38686046 PMC11055990

[15] Yin YX, Ling YH, Wei XL, He CY, Wang BZ, Hu CF, et al. Impact of mature tertiary lymphoid structures on prognosis and therapeutic response of Epstein-Barr virus-associated gastric cancer patients. Front Immunol. 2022;13:973085. doi:10.3389/fimmu.2022.973085; 36591236 PMC9794571

[16] Sautès-Fridman C, Petitprez F, Calderaro J, Fridman WH. Tertiary lymphoid structures in the era of cancer immunotherapy. Nat Rev Cancer. 2019;19(6):307–25. doi:10.1038/s41568-019-0144-6; 31092904

[17] Domblides C, Rochefort J, Riffard C, Panouillot M, Lescaille G, Teillaud JL, et al. Tumor-associated tertiary Lymphoid structures: from basic and clinical knowledge to therapeutic manipulation. Front Immunol. 2021;12:698604. doi:10.3389/fimmu.2021.698604; 34276690 PMC8279885

[18] Lin Q, Tao P, Wang J, Ma L, Jiang Q, Li J, et al. Tumor-associated tertiary lymphoid structure predicts postoperative outcomes in patients with primary gastrointestinal stromal tumors. Oncoimmunology. 2020;9(1):1747339. doi:10.1080/2162402x.2020.1747339; 32313726 PMC7153826

[19] Lu Y, Zhao Q, Liao JY, Song E, Xia Q, Pan J, et al. Complement signals determine opposite effects of B cells in chemotherapy-induced immunity. Cell. 2020;180(6):1081–97.e24. doi:10.1016/j.cell.2020.02.015; 32142650

[20] Xie J, Lin X, Deng X, Tang H, Zou Y, Chen W, et al. Cancer-associated fibroblast-derived extracellular vesicles: regulators and therapeutic targets in the tumor microenvironment. Cancer Drug Resist. 2025;8:2. doi:10.20517/cdr.2024.152; 39935427 PMC11810458

[21] Subramanian A, Tamayo P, Mootha VK, Mukherjee S, Ebert BL, Gillette MA, et al. Gene set enrichment analysis: a knowledge-based approach for interpreting genome-wide expression profiles. Proc Natl Acad Sci U S A. 2005;102(43):15545–50. doi:10.1073/pnas.0506580102; 16199517 PMC1239896

[22] Stuart T, Butler A, Hoffman P, Hafemeister C, Papalexi E, Mauck WM 3rd, et al. Comprehensive integration of single-cell data. Cell. 2019;177(7):1888–902.e21. doi:10.1016/j.cell.2019.05.031; 31178118 PMC6687398

[23] Zhang M, Hu S, Min M, Ni Y, Lu Z, Sun X, et al. Dissecting transcriptional heterogeneity in primary gastric adenocarcinoma by single cell RNA sequencing. Gut. 2021;70(3):464–75. doi:10.1136/gutjnl-2019-320368; 32532891 PMC7873416

[24] Eisenhauer EA, Therasse P, Bogaerts J, Schwartz LH, Sargent D, Ford R, et al. New response evaluation criteria in solid tumours: revised RECIST guideline (version 1.1). Eur J Cancer. 2009;45(2):228–47. doi:10.1016/j.ejca.2008.10.026; 19097774

[25] Trapnell C, Cacchiarelli D, Grimsby J, Pokharel P, Li S, Morse M, et al. The dynamics and regulators of cell fate decisions are revealed by pseudotemporal ordering of single cells. Nat Biotechnol. 2014;32(4):381–6. doi:10.1038/nbt.2859; 24658644 PMC4122333

[26] Taniguchi CM, Winnay J, Kondo T, Bronson RT, Guimaraes AR, Alemán JO, et al. The phosphoinositide 3-kinase regulatory subunit p85α can exert tumor suppressor properties through negative regulation of growth factor signaling. Cancer Res. 2010;70(13):5305–15. doi:10.1158/0008-5472.Can-09-3399; 20530665 PMC3204358

[27] Allard B, Longhi MS, Robson SC, Stagg J. The ectonucleotidases CD39 and CD73: novel checkpoint inhibitor targets. Immunol Rev. 2017;276(1):121–44. doi:10.1111/imr.12528; 28258700 PMC5338647

[28] Xue G, Wang Z, Zheng N, Fang J, Mao C, Li X, et al. Elimination of acquired resistance to PD-1 blockade via the concurrent depletion of tumour cells and immunosuppressive cells. Nat Biomed Eng. 2021;5(11):1306–19. doi:10.1038/s41551-021-00799-6; 34725506 PMC8595849

[29] Silva LFL, Scholl JN, Weber AF, Dias CK, Pizzato PR, Lima VP, et al. Assessing the impact of CD73 inhibition on overcoming anti-EGFR resistance in glioma cells. Oncol Res. 2025;33(4):951–64. doi:10.32604/or.2024.055508; 40191718 PMC11964884

[30] Teicher BA. Malignant cells, directors of the malignant process: role of transforming growth factor-beta. Cancer Metastasis Rev. 2001;20(1–2):133–43. doi:10.1023/a:1013177011767; 11831642

[31] Chen S, Fan J, Zhang M, Qin L, Dominguez D, Long A, et al. CD73 expression on effector T cells sustained by TGF-β facilitates tumor resistance to anti-4-1BB/CD137 therapy. Nat Commun. 2019;10(1):150. doi:10.1038/s41467-018-08123-8; 30635578 PMC6329764

[32] Trapani JA. The dual adverse effects of TGF-beta secretion on tumor progression. Cancer Cell. 2005;8(5):349–50. doi:10.1016/j.ccr.2005.10.018; 16286241

[33] Harris RJ, Willsmore Z, Laddach R, Crescioli S, Chauhan J, Cheung A, et al. Enriched circulating and tumor-resident TGF-β^+^ regulatory B cells in patients with melanoma promote FOXP3^+^ Tregs. Oncoimmunology. 2022;11(1):2104426. doi:10.1080/2162402x.2022.2104426; 35909944 PMC9336482

[34] Da M, Chen L, Enk A, Ring S, Mahnke K. The multifaceted actions of CD73 during development and suppressive actions of regulatory T Cells. Front Immunol. 2022;13:914799. doi:10.3389/fimmu.2022.914799; 35711418 PMC9197450

[35] Deaglio S, Dwyer KM, Gao W, Friedman D, Usheva A, Erat A, et al. Adenosine generation catalyzed by CD39 and CD73 expressed on regulatory T cells mediates immune suppression. J Exp Med. 2007;204(6):1257–65. doi:10.1084/jem.20062512; 17502665 PMC2118603

[36] Sun Q, Hong Z, Zhang C, Wang L, Han Z, Ma D. Immune checkpoint therapy for solid tumours: clinical dilemmas and future trends. Signal Transduct Target Ther. 2023;8:320. doi:10.1038/s41392-023-01522-4; 37635168 PMC10460796

[37] Allard D, Chrobak P, Allard B, Messaoudi N, Stagg J. Targeting the CD73-adenosine axis in immuno-oncology. Immunol Lett. 2019;205:31–9. doi:10.1016/j.imlet.2018.05.001; 29758241

[38] Jin D, Fan J, Wang L, Thompson LF, Liu A, Daniel BJ, et al. CD73 on tumor cells impairs antitumor T-cell responses: a novel mechanism of tumor-induced immune suppression. Cancer Res. 2010;70:2245–55. doi:10.1158/0008-5472.Can-09-3109; 20179192 PMC2883609

[39] Quan Z, Yang Y, Zheng H, Zhan Y, Luo J, Ning Y, et al. Clinical implications of the interaction between PD-1/PD-L1 and PI3K/AKT/mTOR pathway in progression and treatment of non-small cell lung cancer. J Cancer. 2022;13:3434–43. doi:10.7150/jca.77619; 36313041 PMC9608206

[40] Jiang W, Ouyang X, Li C, Long Y, Chen W, Ji Z, et al. Targeting PI3Kα increases the efficacy of anti-PD-1 antibody in cervical cancer. Immunology. 2023;170:419–38. doi:10.1111/imm.13682; 37469254

[41] Lin CY, Huang KY, Kao SH, Lin MS, Lin CC, Yang SC, et al. Small-molecule PIK-93 modulates the tumor microenvironment to improve immune checkpoint blockade response. Sci Adv. 2023;9(14):eade9944. doi:10.1126/sciadv.ade9944; 37027467 PMC10081850

[42] Wei Y, Yin L, Zhang J, Tang J, Yu X, Wu Z, et al. Heterophyllin B inhibits the malignant phenotypes of gastric cancer cells via CXCR4. Hum Cell. 2023;36(2):676–88. doi:10.1007/s13577-022-00824-z; 36539682

[43] Peng K, Liu Y, Liu S, Wang Z, Zhang H, He W, et al. Targeting MEK/COX-2 axis improve immunotherapy efficacy in dMMR colorectal cancer with PIK3CA overexpression. Cell Oncol. 2024;47(3):1043–58. doi:10.1007/s13402-024-00916-y; 38315285 PMC12974043

[44] De Wispelaere W, Annibali D, Tuyaerts S, Messiaen J, Antoranz A, Shankar G, et al. PI3K/mTOR inhibition induces tumour microenvironment remodelling and sensitises pS6^high^ uterine leiomyosarcoma to PD-1 blockade. Clin Transl Med. 2024;14(5):e1655. doi:10.1002/ctm2.1655; 38711203 PMC11074386

[45] Cui R, Luo Z, Zhang X, Yu X, Yuan G, Li X, et al. Targeting PI3K signaling to overcome tumor immunosuppression: synergistic strategies to enhance cancer vaccine efficacy. Vaccines. 2025;13(3):292. doi:10.3390/vaccines13030292; 40266213 PMC11946485

[46] Lauder SN, Smart K, Bart VMT, Pires A, Scott J, Milutinovic S, et al. Treg-driven tumour control by PI3Kdelta inhibition limits myeloid-derived suppressor cell expansion. Br J Cancer. 2022;127(9):1595–602. doi:10.1038/s41416-022-01917-0; 35986086 PMC9596434

[47] Liu L, Niu L, Zheng X, Xiao F, Sun H, Deng W, et al. PD-L1 expression-related PI3K pathway correlates with immunotherapy efficacy in gastric cancer. Ther Adv Med Oncol. 2023;15:17588359231205853. doi:10.1177/17588359231205853; 37868079 PMC10586003

[48] Yeong J, Goh D, Tan TJ, Tan B, Sivaraj H, Koh V, et al. Early triple-negative breast cancers in a Singapore cohort exhibit high PIK3CA mutation rates associated with low PD-L1 expression. Mod Pathol. 2023;36(4):100056. doi:10.1016/j.modpat.2022.100056; 36788078

[49] Wang S, Liu C, Yang C, Jin Y, Cui Q, Wang D, et al. PI3K/AKT/mTOR and PD-1/CTLA-4/CD28 pathways as key targets of cancer immunotherapy (review). Oncol Lett. 2024;28(6):567. doi:10.3892/ol.2024.14700; 39390982 PMC11465225

[50] Zhang B, Leung PC, Cho WC, Wong CK, Wang D. Targeting PI3K signaling in lung cancer: advances, challenges and therapeutic opportunities. J Transl Med. 2025;23(1):184. doi:10.1186/s12967-025-06144-8; 39953539 PMC11829425

