# Supplementary material for: PIK3R1 as a Gastric Cancer Biomarker Linked to CD73+ Treg-Mediated Immunosuppression
Source: Oncol Res. 2026 Jan 19;34(2):17. doi: 10.32604/or.2025.069453 (PMC12848688; doi:10.32604/or.2025.069453)
Supplement: Supplementary file 1 [file OncolRes-34-69453-s001.docx]

Supplementary Materials

Table S1. Total number of cells for each sample in single-cell RNA sequencing data

| Sample | Number of cells |
| --- | --- |
| GSM4546300 | 3932 |
| GSM4546302 | 4850 |
| GSM4546304 | 6457 |
| GSM4546306 | 4949 |
| GSM4546308 | 6986 |
| GSM4546310 | 13172 |
| GSM4546312 | 7034 |
| GSM4546314 | 6315 |
| GSM4546316 | 7794 |
| GSM4546318 | 7701 |

**Table S2. Signatures of different kinds of cells**

| Cells | Marker Genes |
| --- | --- |
| T cell | CD3D, CD3E, CD3G, CD2, CD69 |
| B cell | CD79A, CD79B |
| NK cell | CD14, CPA3, KIT, FCER1G, FCGR3A |
| Fibroblasts | ACTA2, COL1A2, |
| Endothelial cell | ENG, VWF |
| Epithelial cell | EPCAM, KRT18, KRT8, SFN, KRTCAP3 |
| Malignant gastric epithelial cell | CDC20, MYBL2, UBE2C, CLDN3, MISP, EPCAM, MUC13, LGALS4, VIL1, GPCR5A, REG4, CLDN7, CDKN2A, GDF15, CLDN1, HOXA10 |

**Table S3. Expression levels of CD73, CD8, and Foxp3 in the Sun Yat-sen University Cancer Center validation cohort**

| PIK3R1 | | | | | | | | |
| --- | --- | --- | --- | --- | --- | --- | --- | --- |
|  | | | Tumor cell+ Immune cell | | Tumor cell | | Immune cell | |
|  |  |  | High | Low | High | Low | High | Low |
| CD73, n (%) | Tumor cell + Immune cell | High | 13(41.9) | 46(23.1) | 48(43.6) | 11(9.2) | 9(33.3) | 50(24.6) |
|  |  | Low | 18(58.1) | 153(76.9) | 62(56.4) | 109(90.8) | 18(66.7) | 153(75.4) |
|  |  |  | ***P* = 0.026** | | ***P* = 2.24×10^-9^** | | *P* = 0.331 | |
|  | Tumor cell | High | 27(87.1) | 127(63.8) | 84(76.4) | 70(58.3) | 19(70.4) | 135(66.5) |
|  |  | Low | 4(12.9) | 72(36.2) | 26(23.6) | 50(41.7) | 8(29.6) | 68(33.5) |
|  |  |  | ***P* = 1.04×10^-2^** | | ***P* = 0.004** | | *P* = 0.688 | |
|  | Immune cell | High | 17(54.8) | 65(32.7) | 54(49.1) | 28(23.3) | 12(44.4) | 70(34.5) |
|  |  | Low | 14(45.2) | 134(67.3) | 56(50.9) | 92(76.7) | 15(55.6) | 133(65.5) |
|  |  |  | ***P* = 0.016** | | *P* = 0.310 | | *P* = 0.310 | |
| Foxp3, n (%) |  | High | 17(54.8) | 64(32.2) | 45(40.9) | 36(30.0) | 15(55.6) | 66(32.5) |
|  |  | Low | 14(45.2) | 135(67.8) | 65(59.1) | 84(70.0) | 12(44.4) | 137(67.5) |
|  |  |  | ***P* = 0.014** | | ***P* = 0.019** | | ***P* = 0.019** | |
| CD8, n (%) |  | High | 9(29.0) | 61(30.7) | 34(30.9) | 36(30.0) | 11(40.7) | 59(29.1) |
|  |  | Low | 22(71.0) | 138(69.3) | 76(69.1) | 84(70.0) | 16(59.3) | 144(70.9) |
|  |  |  | *P* = 0.855 | | *P* = 0.881 | | *P* = 0.215 | |

Note: Bold values denote statistical significance at the *P* value < 0.05 level; PIK3R1, Phosphoinositide-3-Kinase Regulatory Subunit 1; CD73, cluster of differentiation 73; Foxp, forkhead box protein P3; CD8, cluster of differentiation 8.

**Table S4. Univariate and multivariate analyses of overall survival in patients with gastric cancer**

| Clinicopathological Features | TCGA cohort | | | | SYSUCC cohort | | | |
| --- | --- | --- | --- | --- | --- | --- | --- | --- |
|  | Univariate analysis | | Multivariate analysis | | Univariate analysis | | Multivariate analysis | |
|  | HR (95%CI) | *P* value | HR (95% CI) | *P* value | HR (95% CI) | *P* value | HR (95% CI) | *P* value |
| Age | 1.02(1.01-1.04) | **5.50×10^-3^** | 1.03(1.00-1.05) | **2.70×10^-2^** | 1.04(1.01-1.07) | 2.91×10^-3^ | 1.02(0.98-1.06) | 0.38 |
| TNM stage |  |  |  |  |  |  |  |  |
| I | Reference |  |  |  | Reference |  |  |  |
| II&III&IV | 2.04(1.06-3.90) | **3.19×10^-2^** | 2.32(0.89-6.05) | 8.50×10^-2^ | 16.38(2.26-118.76) | 5.70×10^-3^ | / | 0.99 |
| M stage |  |  |  |  |  |  |  |  |
| M0 | Reference |  |  |  | Reference |  |  |  |
| M1 | 2.09(1.15-3.79) | **1.55×10^-2^** |  |  | 3.74(1.95-7.18) | **1.00×10^-4^** | 1.14(0.57-2.28) |  |
| N stage |  |  |  |  |  |  |  |  |
| N0 | Reference |  |  |  | Reference |  |  |  |
| N1&N2&N3 | 1.81(1.17-2.80) | **7.80×10^-3^** |  |  | 8.07(2.51-25.97) | 5.00×10^-4^ | 1.99(0.42-9.32) |  |
| T stage |  |  |  |  |  |  |  |  |
| T1&T2 | Reference |  |  |  | Reference |  |  |  |
| T3&T4 | 1.82(1.15-2.82) | **0.01** |  |  | 7.37(2.55-19.80) | 4.00×10^-4^ | 2.59(0.75-8.87) |  |
| Therapy evaluation | | | | | | | | |
| PD | Reference |  |  |  | Reference |  |  |  |
| CR&PR&SD | 0.32(0.18-0.55) | **1.00×10^-4^** | 0.28(0.16-0.52) | **3.55×10^-5^** | 0.04(0.02-0.07) | 2.00×10^-16^ | 0.07(0.03-0.17) | **9.51×10^-10^** |
| CD73 |  |  |  |  |  |  |  |  |
| High | Reference |  |  |  | Reference |  |  |  |
| Low | 0.52(0.37-0.74) | **2.00×10^-4^** | 0.63(0.38-1.04) | 0.07 | 0.19(0.11-0.34) | 1.17×10^-8^ | 0.63(0.32-1.23) | 0.18 |
| PIK3R1 |  |  |  |  |  |  |  |  |
| High | Reference |  |  |  | Reference |  |  |  |
| Low | 0.5(0.34-0.74) | **5.00×10^-4^** | 0.38(0.22-0.67) | **7.09×10^-4^** | 0.25(0.14-0.45) | 4.46×10^-6^ | 0.39(0.21-0.74) | **4.00×10^-3^** |

Note: Bold values denote statistical significance at the *P* value<0.05 level. TCGA, The Cancer Genome Atlas; SYSUCC, Sun Yat-sen University Cancer Center; HR, Hazard Ratio; CI, Confidence Interval; PD, Progressive Disease; CR, Complete Response; PR, Partial Response; SD, Stable Disease; CD73 cluster of differentiation 73; PIK3R1, Phosphoinositide-3-Kinase Regulatory Subunit 1.

**Figure S1** PIK3R1 expression in gastric cancer cell lines from the DepMap data.

**Figure S2** MKN45 was treated with PIK3R1 knockdown or overexpression. HGC27 was overexpressed PIK3R1. The PIK3R1 mRNA level was significantly reduced in PIK3R1 knockdown MKN45 (****P* < 0.001) (A). And the PIK3R1 mRNA levels of MKN45 and HGC27, which were treated with PIK3R1 overexpression, were significantly upregulated (****P* < 0.001) (A, B). Western blots also showed differences in their protein expression levels, and knockdown of PIK3R1 led to decreased levels of p-AKT and CD73 overexpression leads to elevated levels of p-AKT and CD73 (C).

**Figure S3** Kaplan-Meier survival curves based on combined PIK3R1, CD8, and CD73 expression levels in different groups in the Sun Yat-sen University Cancer Center (SYSUCC) and The Cancer Genome Atlas (TCGA) cohorts. TCGA cohort: No statistically significant difference was observed in overall survival (OS) between the CD8high and CD8low groups (*P* = 0.059) (A). Patients with gastric cancer (GC) with PIK3R1^high^-CD8^high^ exhibited the shortest OS time (*P* = 0.004) (B). Patients with GC in the CD73low group had increased OS time compared to those in the CD73high group (*P* = 0.012) (C). Patients in the PIK3R1^low^-CD73low group had the longest OS time, while those in the PIK3R1 high-CD73 high group had the shortest OS time (*P* = 7.42 × 10^-5^) (D). SYSUCC cohort: The OS of patients with GC was higher in the CD8high group than that of those in the CD8low group (*P* = 0.040) (E). The PIK3R1^low^-CD8^high^ group had the longest OS time, while the PIK3R1^high^-CD8^low^ group had the shortest OS time (*P* = 1.86 × 10^-6^) (F). The OS time of patients with GC was greater in the CD73low group than that of those in the CD73high group (*P* = 1.98 × 10^-10^) (G). The PIK3R1^low^-CD73^low^ group had the longest OS time, while the PIK3R1^high^-CD73^high^ group had the shortest OS time (*P* = 2.66 × 10^-15^) (H).

**Figure S4** Unedited original WB images.

Supplement file S1:

**(1) The Sequences of PIK3R1-shRNA.**

| Name | Sequence (5'-3') |
| --- | --- |
| shNC | CCTAAGGTTAAGTCGCCCTCG |
| shPIK3R1 | TTCATCGAGATGGGAAATATG |

**(2) The sequence of PIK3R1 overexpression.**

Name: hPIK3R1 [NM_181523.3]

Type: CDS

Sequence (Length: 2175 bp)

ATGAGTGCTGAGGGGTACCAGTACAGAGCGCTGTATGATTATAAAAAGGAAAGAGAAGAAGATATTGACTTGCACTTGGGTGACATATTGACTGTGAATAAAGGGTCCTTAGTAGCTCTTGGATTCAGTGATGGACAGGAAGCCAGGCCTGAAGAAATTGGCTGGTTAAATGGCTATAATGAAACCACAGGGGAAAGGGGGGACTTTCCGGGAACTTACGTAGAATATATTGGAAGGAAAAAAATCTCGCCTCCCACACCAAAGCCCCGGCCACCTCGGCCTCTTCCTGTTGCACCAGGTTCTTCGAAAACTGAAGCAGATGTTGAACAACAAGCTTTGACTCTCCCGGATCTTGCAGAGCAGTTTGCCCCTCCTGACATTGCCCCGCCTCTTCTTATCAAGCTCGTGGAAGCCATTGAAAAGAAAGGTCTGGAATGTTCAACTCTATACAGAACACAGAGCTCCAGCAACCTGGCAGAATTACGACAGCTTCTTGATTGTGATACACCCTCCGTGGACTTGGAAATGATCGATGTGCACGTTTTGGCTGACGCTTTCAAACGCTATCTCCTGGACTTACCAAATCCTGTCATTCCAGCAGCCGTTTACAGTGAAATGATTTCTTTAGCTCCAGAAGTACAAAGCTCCGAAGAATATATTCAGCTATTGAAGAAGCTTATTAGGTCGCCTAGCATACCTCATCAGTATTGGCTTACGCTTCAGTATTTGTTAAAACATTTCTTCAAGCTCTCTCAAACCTCCAGCAAAAATCTGTTGAATGCAAGAGTACTCTCTGAAATTTTCAGCCCTATGCTTTTCAGATTCTCAGCAGCCAGCTCTGATAATACTGAAAACCTCATAAAAGTTATAGAAATTTTAATCTCAACTGAATGGAATGAACGACAGCCTGCACCAGCACTGCCTCCTAAACCACCAAAACCTACTACTGTAGCCAACAACGGTATGAATAACAATATGTCCTTACAAGATGCTGAATGGTACTGGGGAGATATCTCGAGGGAAGAAGTGAATGAAAAACTTCGAGATACAGCAGACGGGACCTTTTTGGTACGAGATGCGTCTACTAAAATGCATGGTGATTATACTCTTACACTAAGGAAAGGGGGAAATAACAAATTAATCAAAATATTTCATCGAGATGGGAAATATGGCTTCTCTGACCCATTAACCTTCAGTTCTGTGGTTGAATTAATAAACCACTACCGGAATGAATCTCTAGCTCAGTATAATCCCAAATTGGATGTGAAATTACTTTATCCAGTATCCAAATACCAACAGGATCAAGTTGTCAAAGAAGATAATATTGAAGCTGTAGGGAAAAAATTACATGAATATAACACTCAGTTTCAAGAAAAAAGTCGAGAATATGATAGATTATATGAAGAATATACCCGCACATCCCAGGAAATCCAAATGAAAAGGACAGCTATTGAAGCATTTAATGAAACCATAAAAATATTTGAAGAACAGTGCCAGACCCAAGAGCGGTACAGCAAAGAATACATAGAAAAGTTTAAACGTGAAGGCAATGAGAAAGAAATACAAAGGATTATGCATAATTATGATAAGTTGAAGTCTCGAATCAGTGAAATTATTGACAGTAGAAGAAGATTGGAAGAAGACTTGAAGAAGCAGGCAGCTGAGTATCGAGAAATTGACAAACGTATGAACAGCATTAAACCAGACCTTATCCAGCTGAGAAAGACGAGAGACCAATACTTGATGTGGTTGACTCAAAAAGGTGTTCGGCAAAAGAAGTTGAACGAGTGGTTGGGCAATGAAAACACTGAAGACCAATATTCACTGGTGGAAGATGATGAAGATTTGCCCCATCATGATGAGAAGACATGGAATGTTGGAAGCAGCAACCGAAACAAAGCTGAAAACCTGTTGCGAGGGAAGCGAGATGGCACTTTTCTTGTCCGGGAGAGCAGTAAACAGGGCTGCTATGCCTGCTCTGTAGTGGTGGACGG

Supplement file S2:

**Major reagents with catalog ID and supplier.**

| **Reagent** | **Catalog ID** | **Supplier** |
| --- | --- | --- |
| Gateway® BP Clonase™ II Enzyme Mix | 11789020 | Invitrogen |
| Gateway® LR Clonase™ II Plus Enzyme Mix | 12538120 | Invitrogen |
| PrimeSTAR™ HS DNA Polymerase | R010Q | Takara |
| Plasmid small extraction kit | DP103 | TiANGEN |
| Taq DNA Polymerase | EP0404 | Fermentas |
| dNTP Mix | R1121 | Fermentas |
| GeneRuler™ 100bp DNA Ladder | SM0241/SM0242 | Fermentas |
| Neon™ Kit | MPK1096 | Invitrogen |
| AceQ® Universal SYBR qPCR Master Mix | Q511-02/03 | Novizan |
| RNAprep pure Tissue Kit | DP431 | TIANGEN |
| HiScript III 1st Strand cDNA Synthesis Kit (+gDNA wiper) | R312-02 | TIANGEN |
